# Application of metallic guides and reverse scanning for full‐mouth rehabilitation using implant‐supported prostheses: A case report

**DOI:** 10.1111/jopr.70026

**Published:** 2025-08-22

**Authors:** Ahlam A. Alhazmi, Mazin Talal Alharbi, Anas Lahiq, Lama Saleh AlMarshoud, Ihab Tawfiq Mitwalli, Rayan Asali, Amr Ahmed Azhari, Walaa Magdy Ahmed

**Affiliations:** ^1^ Department of Prosthodontics Jazan Specialist Dental Center, Jazan Health Cluster Jazan Saudi Arabia; ^2^ Division of Prosthodontics King Fahad Armed Forces Hospital, Ministry of Defense Jeddah Saudi Arabia; ^3^ Department of Prosthodontics Aseer Dental Specialized Center Abha Saudi Arabia; ^4^ Division of Periodontics King Fahad Armed Forces Hospital, Ministy of Defense Jeddah Saudi Arabia; ^5^ Department of Restorative Dentistry, Faculty of Dentistry King Abdulaziz University Jeddah Saudi Arabia

**Keywords:** complete arch, digital dentistry, guided implant surgery, reverse scan

## Abstract

Advances in dental implantology, such as immediate loading protocols, digital planning, and improved biomaterials, have revolutionized the treatment of edentulous patients by offering faster and more predictable outcomes. This case report describes a complete digital workflow for maxillary and mandibular implant‐supported full‐arch prostheses in an edentulous patient with nonuniform bone shape, limited interarch space, and excessive gingival display. A full‐arch immediate loading protocol, including a fixation base, a scalloped bone‐reduction guide for preserving the interdental bone, an osteotomy guide, and provisional prostheses, was digitally designed and fabricated. The reverse scan technique using laboratory analog was used for fabricating definitive zirconia prostheses. The reverse scan technique integrates digital technology with clinical practice to improve the accuracy of full‐arch implant restorations.

The prevalence of edentulism is rising globally,^1^ driven by factors such as aging populations, chronic diseases, and disparities in access to dental care. This has led to a growing demand for full‐arch implant rehabilitation as patients increasingly seek durable, esthetic, and functional solutions to restore their oral health.^2^ Advances in dental implantology, such as immediate loading protocols, digital planning, and improved biomaterials, have revolutionized the treatment of edentulous patients by offering faster and more predictable outcomes. Proper execution of phased guided implant surgical protocols with adequate planning using computer‐aided design and computer‐aided manufacturing (CAD‐CAM) can improve the accuracy, efficiency, and predictability of complex full‐mouth implant rehabilitation.^3^ However, digital workflows in implant prosthodontics are challenging owing to the absence of nonmobile anatomical landmarks in edentulous patients, which poses a challenge in registering and aligning data.^4^ Nonetheless, digital workflows reduce postoperative complications and increase patient satisfaction while maintaining the long‐term predictability of treatment outcomes.^5^ Several procedures for full‐arch implant rehabilitation, such as 3‐dimensional (3D) imaging incorporating surface scans, 3D smile design, and virtual mock‐up, can be performed digitally.^6^ Subsequently, immediate loading can be achieved with high accuracy using digitally designed provisional restorations.^3^

Herein, a fully digital workflow is described, including digital planning and fabrication of implant‐supported prostheses and guided reduction of excess bone in a patient with nonuniform maxillary and mandibular bone shapes and limited interarch space. In addition, a reverse scan technique for capturing the maxillary and mandibular arch implant positions and interocclusal record is described.

## CLINICAL REPORT

### Treatment planning

**FIGURE 1:**
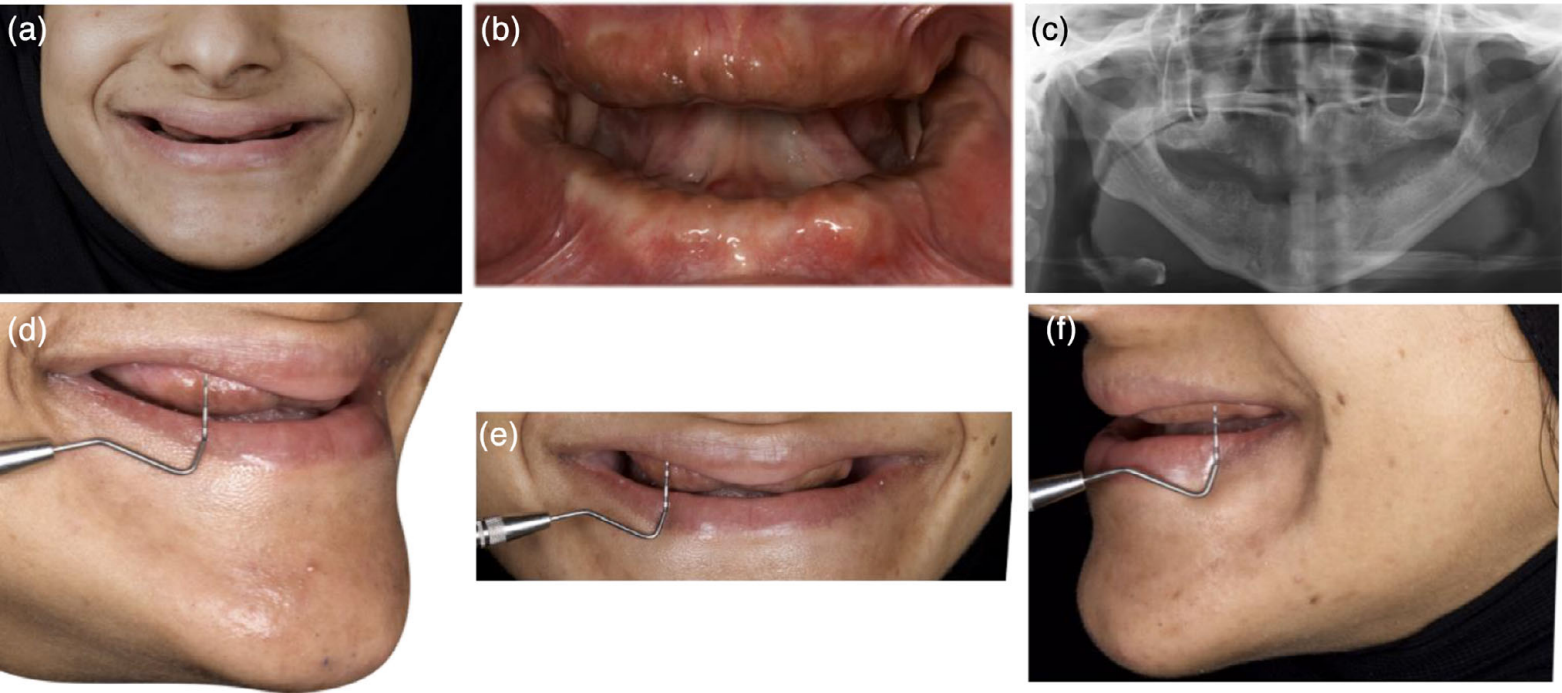
(a) Preoperative extraoral photograph; (b) intraoral photographs; and (c) orthopantomogram showing the edentulous maxilla and mandible. Extraoral photographs showing excessive bone display with nonuniform bone shape: (d) right profile view, (e) frontal view, and (f) left profile view.

A 26‐year‐old woman with no relevant medical or family history presented to the prosthodontic clinic with a chief complaint of not being able to eat properly or smile with confidence. The patient had all her teeth extracted due to generalized periodontitis (Periodontitis stage III grade C). Intraoral examination revealed edentulous maxillary and mandibular arches (Figure 1) with nonuniform bone shapes, limited interarch space, a high smile line (Figure 1), and a normal lip length with hypermobile lips. The patient provided informed consent for implant‐supported fixed dental prostheses treatment in both arches.

**FIGURE 2:**
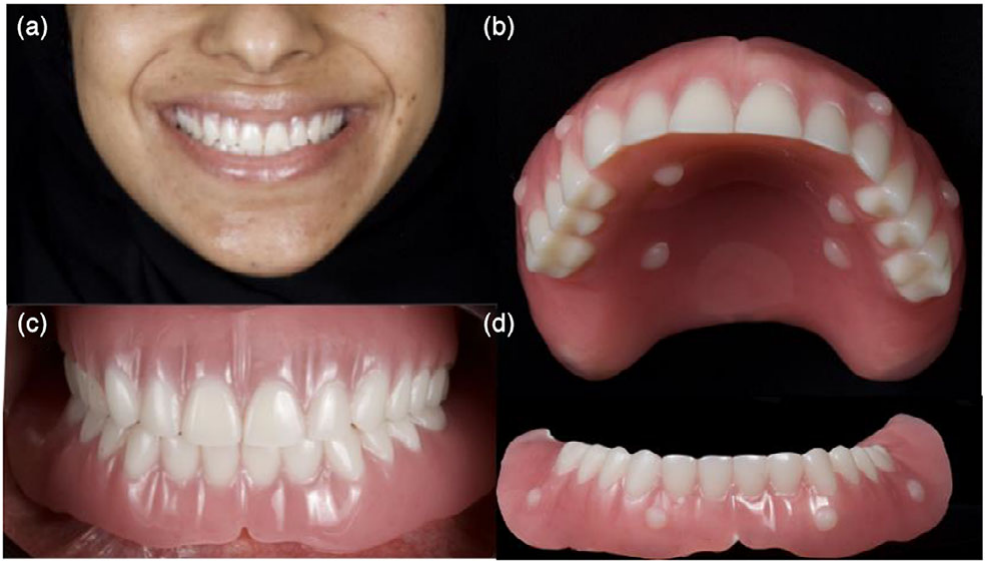
(a) Extraoral and (b) intraoral photographs showing complete dentures in situ. Markers on the dentures for dual scan: (c) maxillary and (d) mandibular.

**FIGURE 3:**
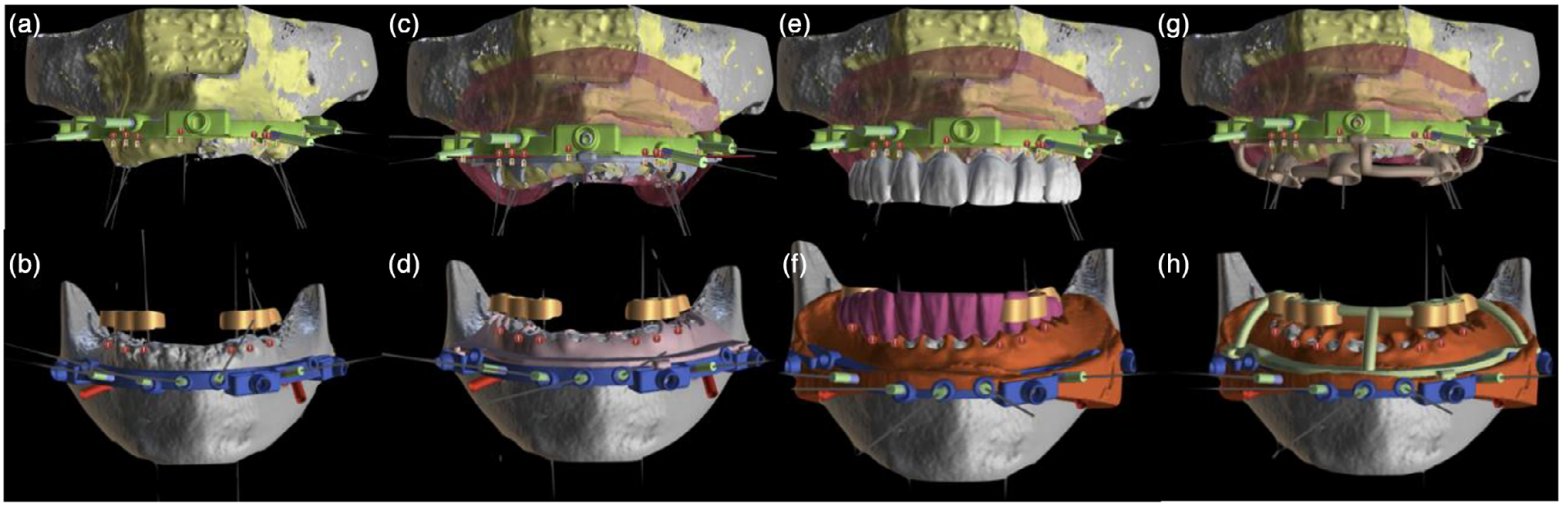
Fixation base design and the positions of the guide pins: (a) maxillary, (b) mandibular. Design of the scalloped bone‐reduction guide: (c) maxillary, (d) mandibular. Provisional prosthesis design: (e) maxillary, (f) mandibular. Implant placement surgical guide design: (g) maxillary, (h) mandibular.

First, maxillary and mandibular complete dentures were fabricated conventionally using heat‐cure acrylic resin (Figure 2). The dentures were evaluated for esthetics, phonetics, vertical dimension of occlusion, and lip support. Next, fiducial markers were placed on the dentures, and cone‐beam computed tomography (CBCT) was performed using a dual‐scan protocol (Figure 2). Based on the scan data, seven maxillary and six mandibular implants were digitally planned (Figure 3) and placed using a fully guided surgical protocol. To achieve the optimum prosthetic outcome, the number and distribution of implants were determined based on the full‐arch immediate loading protocol recommended by the International Team of Implantology Consensus 2017.^7^

### Surgical phases

Straumann (Switzerland) bone‐level implants were planned at all sites. In the maxillary arch, narrow NC platform (3.3 × 10) and regular CrossFit RC parallel implants (4.1 × 10) were planned. In the mandibular arch, regular CrossFit RC parallel implants (4.8 × 8) were planned. The design sequence was as follows. First, fixation bases were designed, and the positions of the 1.3 mm guide pins were determined (Figure 3). Second, scalloped bone‐reduction guides were designed to preserve the interdental bone (Figure 3). Third, the provisional prostheses were designed according to the planned bone reduction (Figure 3). Finally, the osteotomy guides were designed (Figure 3).

**FIGURE 4:**
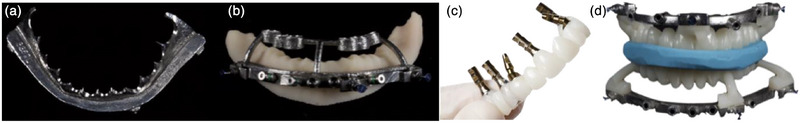
(a) Milled scalloped bone‐reduction guide; (b) milled osteotomy guide; (c) 3D‐printed provisional prosthesis; (d) milled provisional prostheses with interocclusal record.

The fixation, bone reduction (Figure 4), and osteotomy guides (Figure 4) were milled in chrome metal, and the provisional prostheses were printed using resin for immediate loading on the day of surgery (Figure 4) along with the inter‐occlusal record made of polyvinyl siloxane (PVS) Occlufast Rock Zhermack (Italy), to ensure precise positioning of the surgical guides (Figure 4).

**FIGURE 5:**
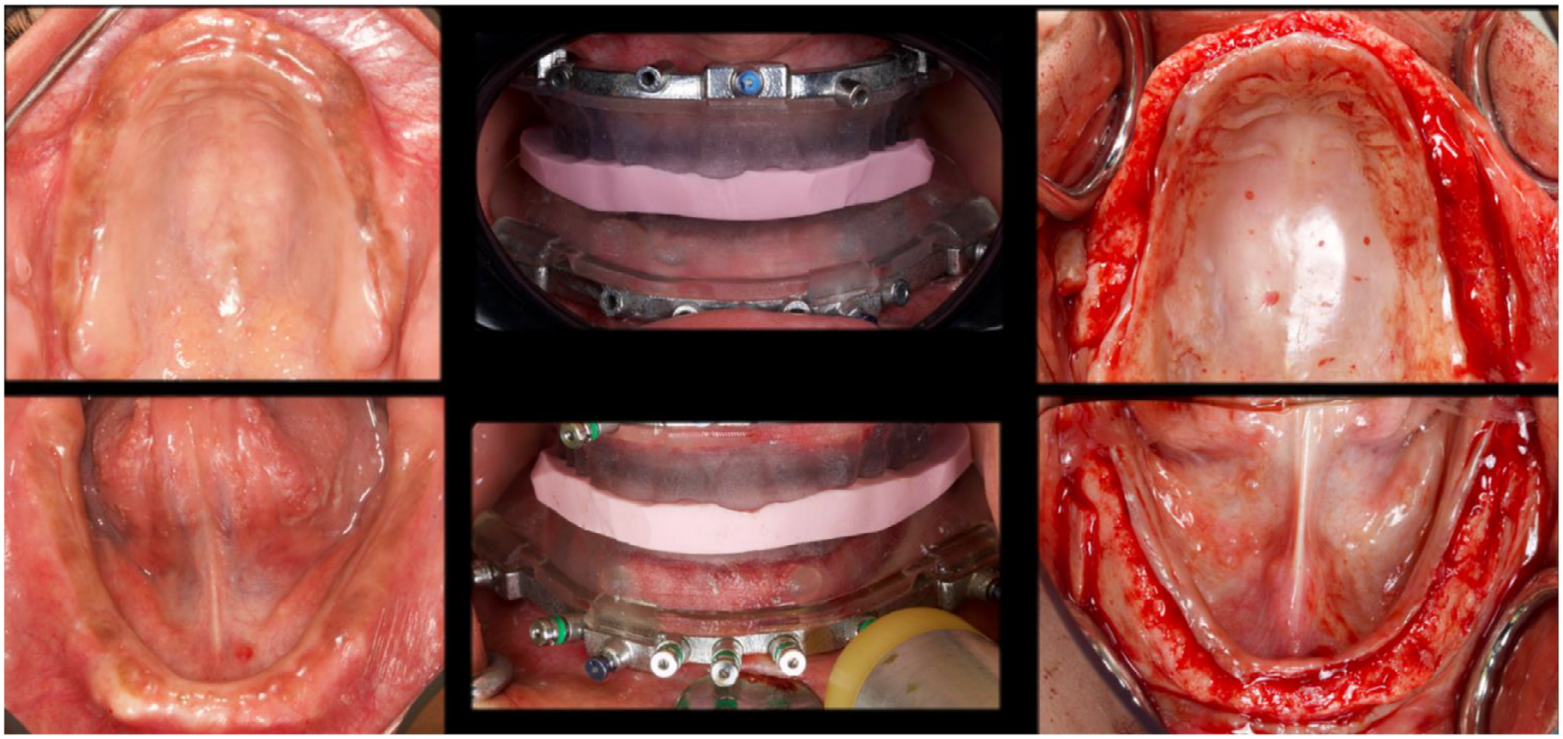
Intraoperative photographs showing maxillary and mandibular insertion of the fixation guides using the interocclusal record.

**FIGURE 6:**
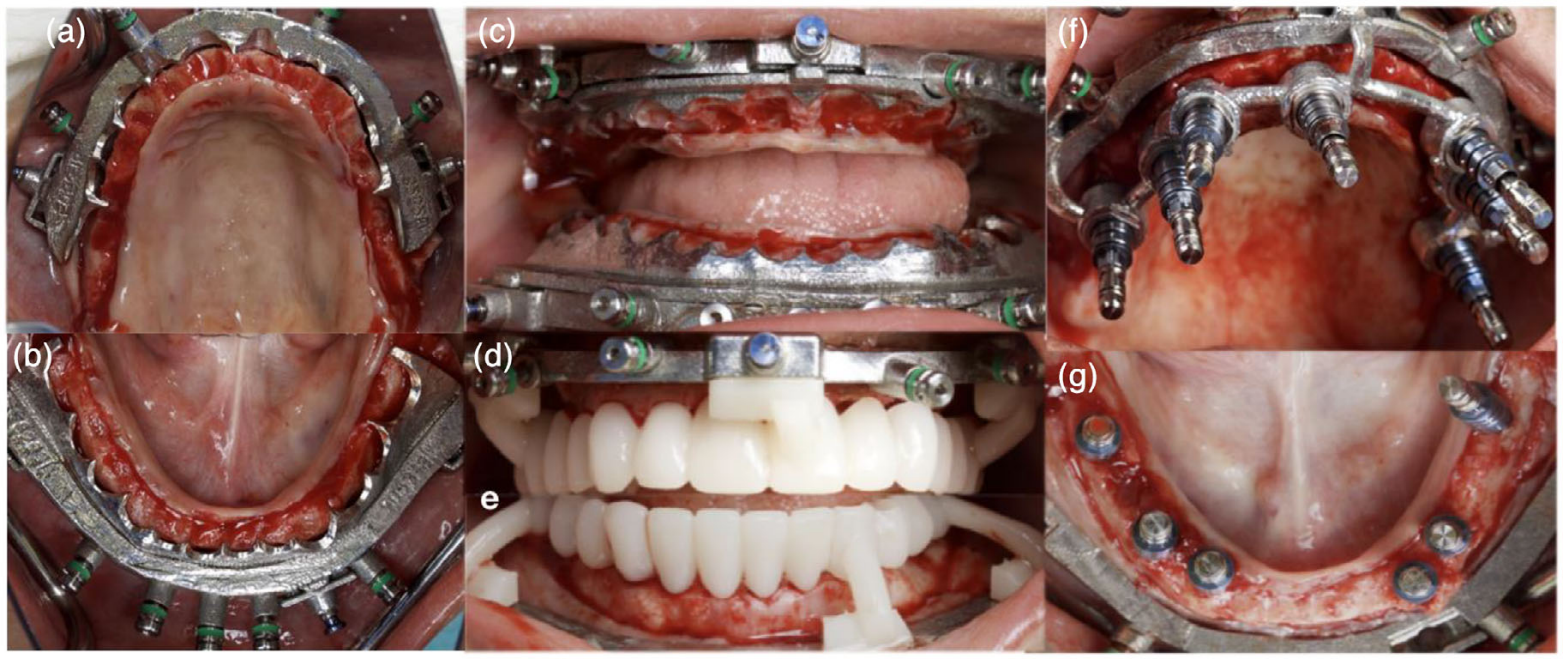
Metallic scalloped bone‐reduction guides in situ: (a) maxillary occlusal view, (b) mandibular occlusal view, (c) frontal view. Osteotomy verification using the provisional prostheses: (d) maxillary, (e) mandibular. Implant placement using the metallic surgical guides: (f) maxillary, (g) mandibular.

**FIGURE 7:**
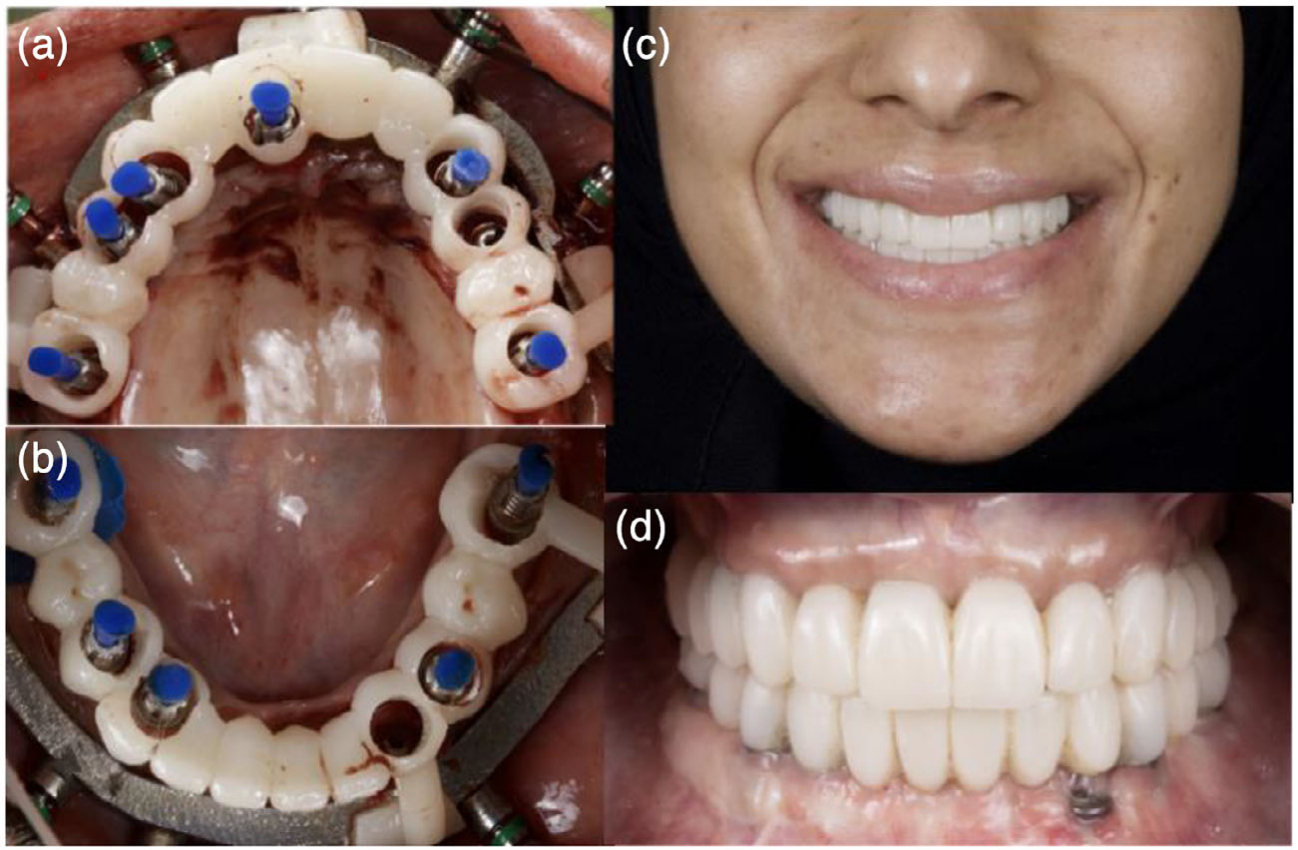
Provisional prostheses placed using the guide: (a) maxillary, (b) mandibular, (c) extraoral photograph showing the provisional prostheses, and (d) intraoral photograph showing the provisional prostheses.

First, the maxillary and mandibular fixation bases with the inter‐occlusal record to ensure stable positioning during insertion of the fixation pins were delicately inserted (Figure 5). Next, the scalloped bone‐reduction guide was used to precisely shape the alveolar ridge (Figure 6). After verifying proper bone reduction using the provisional prostheses (Figure 6), implants were placed using the surgical guides (Figure 6); all implants achieved adequate primary stability with an insertion torque > 35 Ncm. Therefore, Straumann straight multiunit abutments were placed (Figure 6) and torqued to 30 Ncm, followed by immediate loading using the provisional prostheses (Figure 6). The selection of abutments was based on the prosthetic requirements and soft tissue depth in each region to ensure optimal emergence profile, screw access, and passive fit of the prostheses. The 3D‐oriented provisional prostheses were fabricated using NextDent C&B MFH and a NextDent 3D printer (the Netherlands). The provisional prostheses were relined chairside using a rubber dam as a barrier and polymethyl methacrylate (PMMA) as a material (Temporary Bridge Resin, Dentsply, USA) (Figure 7).

Two implants were not immediately loaded in the provisional prosthesis due to inadequate primary stability at the time of placement. As a result, they were excluded from the initial loading protocol to ensure proper osseointegration and long‐term success. Additionally, chairside adjustments using composite restorations were performed to ensure maximum intercuspation of the interim prostheses in centric occlusion, and optimum esthetics, phonetics, and vertical dimension of occlusion were verified. Extra‐ and intraoral photographs (Figure 7) and an orthopantomogram were obtained on the day of surgery after immediate loading.

### Fabrication of the prostheses

**FIGURE 8:**
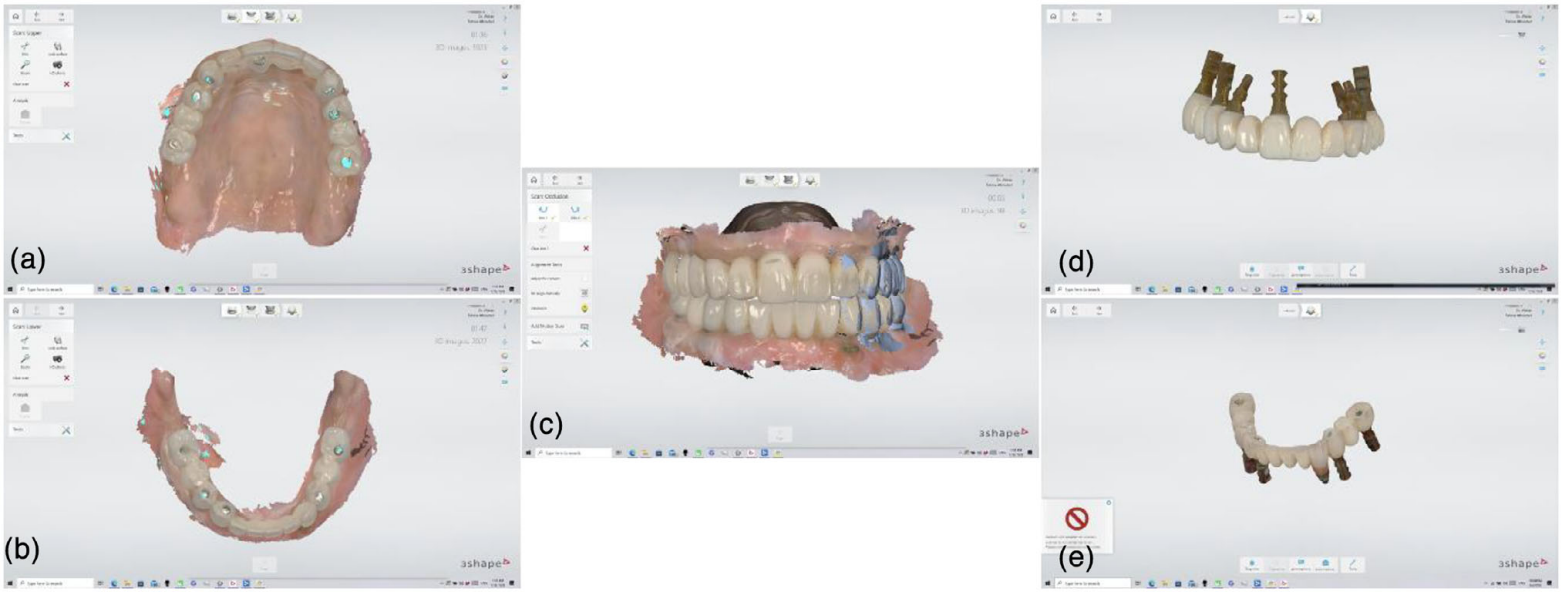
Intraoral scans: (a) maxillary provisional, (b) mandibular provisional, and (c) interocclusal record. Extraoral scans of the provisional restorations after positioning the implant analogs: (d) maxillary, (e) mandibular.

**FIGURE 9:**
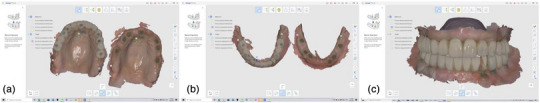
Matching the intraoral scans for each arch: (a) maxillary, (b) mandibular, (c) interocclusal record.

**FIGURE 10:**
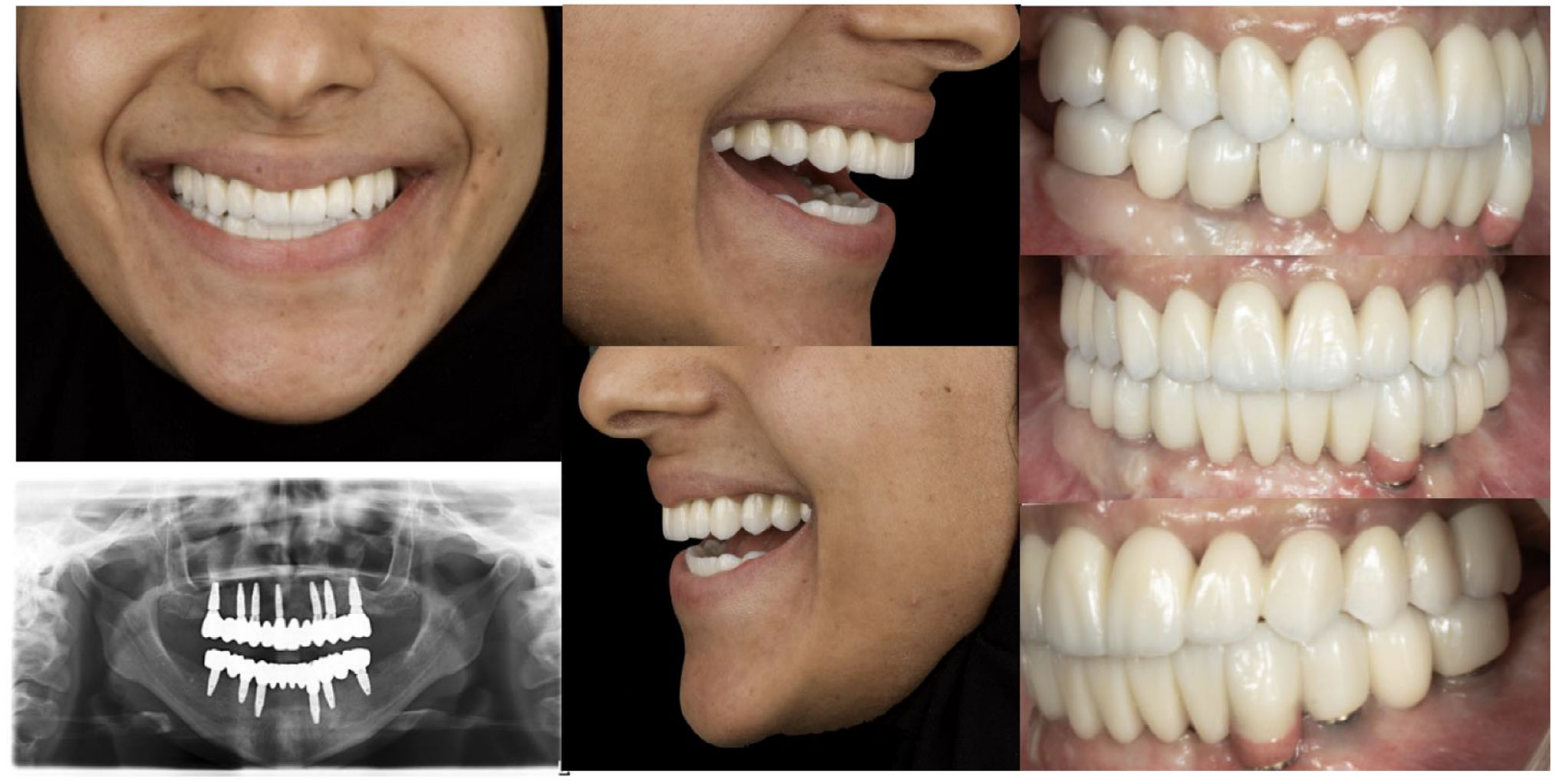
Postoperative extraoral photographs: frontal view, right profile, left profile; postoperative orthopantomogram; and intraoral photographs after delivery of the final milled monolithic zirconia prosthesis.

After allowing 3 months for healing and implant osteointegration, the reverse scan technique proceeded using laboratory implant analogs. The provisional occlusion was recorded using an intraoral scanner (TRIOS 5, 3Shape, Copenhagen, Denmark) (Figure 8). Thereafter, the maxillary and mandibular provisional prostheses were removed, and laboratory implant analogs were attached to them. The assemblies were scanned extraorally to capture the maxillary and mandibular implant positions (Figure 8). All recorded scans were aligned using digital design software (v2.4.3, Medit Design, Medit, Korea) (Figure 9), and the final prostheses, matching the provisionals, were milled in IPS ZirCAD Prime zirconia (Ivoclar, Schaan, Liechtenstein) (Figure 10). The final prostheses were fabricated from monolithic zirconia and cemented to abutment‐level titanium bases (Ti‐bases), and no metal framework was used under the zirconia. Various techniques, such as the screw resistance test, periapical radiographs, and occlusal checks, were used to ensure accurate placement.

Following occlusal evaluation and necessary adjustments, the patient expressed complete satisfaction with the esthetics, function, and comfort of the prostheses. Pink porcelain was incorporated into the final mandibular prosthesis to restore the natural appearance of the gingival architecture, particularly in the canine region where there was soft tissue recession. This esthetic modification was necessary to achieve a harmonious and lifelike result, despite implant placement following the planned protocol.

A postoperative orthopantomogram (Figure 10) and extra‐ and intraoral photographs were obtained after the patient was fully satisfied (Figure 10). The patient was followed up after 24 h, and an occlusal splint was provided. Regular follow‐ups were performed at 3, 6, 12, and 18 months after delivery of the prostheses.

## DISCUSSION

Adherence to appropriate guidelines is crucial to ensure successful implant procedures with long‐lasting results. In particular, maintaining interdental bone integrity is of utmost importance during reduction of excessive bone. The scalloped guide described herein can be used to precisely perform osteotomy for reduction of excessive bone in patients undergoing full‐arch implant‐supported rehabilitation with immediate loading.

Immediate loading of provisional prostheses in full‐arch implant cases provides several benefits for both patients and clinicians, such as improved patient satisfaction and comfort, esthetics, and function. Patients receive functional and esthetic provisional prostheses on the day of surgery, avoiding edentulism during the healing period. In addition to reduced treatment time, immediate restorations provide psychological benefits, such as improved confidence and morale, and enhance quality of life, as patients can smile and eat soft foods early.

Furthermore, immediate provisionalization eliminates the prolonged waiting period between implant placement and prosthesis delivery and enhances efficiency by reducing the number of clinical appointments and chairside time. In addition, the provisional prosthesis improves the preservation of the soft‐tissue and bone contours by providing immediate support to the soft tissues, guiding the emergence profile, and promoting optimal esthetic outcomes. Moreover, it minimizes alveolar bone resorption, maintains tissue architecture, and ⁠facilitates functional loading, which can stimulate osseointegration through functional forces, contributing to implant stabilization and ⁠improved healing. By protecting the surgical site, the provisional prosthesis reduces the risk of trauma and contamination, ensuring better healing outcomes. Furthermore, provisional prostheses enhance patient communication and serve as a diagnostic tool, allowing clinicians to assess and adjust the occlusion, esthetics, phonetics, and patient comfort before fabricating the definitive prosthesis.

The reverse scan technique^8^ is a novel approach used to accurately capture the occlusal records and locations of maxillary and mandibular full‐arch implants. This dual‐scanning method of capturing the implant analog extraorally and the soft tissue and provisionals intraorally, and aligning them digitally, offered an extensive perspective on the spatial relation between the arches, improved the precision of the definitive prostheses, and allowed for integration of occlusal records while scanning the maxillary and mandibular provisionals and soft tissues. This integration is essential for achieving the maximum functional and aesthetic results.

Guided surgery offers high precision but requires meticulous planning, careful execution, and adaptability. Many challenges can affect the accuracy of guided placement of the implants. Technical challenges include guide manufacturing errors, drill tolerance, tool wear or deformation, planning‐related factors like incorrect angulation, CBCT scan artifacts, and inadequate data integration.^9^, ^10^ Surgical challenges in guided surgery include guide stability issues, soft tissue interference, bone density variations, surgeon experience, and patient‐specific factors like anatomical variations, bone quality or volume, and jaw movements. Intraoperative adjustments may involve avoiding anatomical structures, insufficient primary stability, and soft tissue considerations to optimize prosthetic outcomes or ensure better soft tissue healing around the implant. Environmental and equipment factors include limited access in posterior regions, sterility or contamination issues, and human factors like fatigue or stress and poor communication between surgical team members.^9^, ^10^ Understanding and addressing these factors can help minimize deviations and improve outcomes in implant dentistry.

Patient‐centered considerations play a crucial role in selecting an implant‐supported fixed dental prosthesis (FPD), particularly the FP1 design. The patient in this case had compromised teeth due to generalized periodontitis, which were extracted to form edentulous arches. The bone quality and quantity were sufficient for implant placement, ensuring a long‐term, stable solution. An implant‐supported prosthesis after bone reduction provides superior support, stability, and longevity compared to removable prostheses or implant‐assisted options. An FP1 design closely mimics natural teeth, offering better esthetics and confidence for the patient. It requires less restorative space compared to designs with gingival components, making it a preferred choice when adequate restorative space exists but bone reduction is still necessary. FP1 designs also offer better hygiene access and a closer resemblance to natural teeth, enhancing patient satisfaction.

## CONCLUSION

The reverse scan technique is a notable development in dental implantology that integrates digital technology with clinical practice to improve the precision of full‐arch implant restorations. This approach results in reliable and effective implant‐supported restorations.

## CONFLICT OF INTEREST STATEMENT

The authors declare no conflicts of interest.

## References

[1] McKenna G , Tsakos G , Burke F , Brocklehurst P . Managing an ageing population: challenging oral epidemiology. Prim Dent J. 2020;9(3):14–17. 10.1177/2050168420943063 32940594

[2] Lee DJ , Saponaro PC . Management of edentulous patients. Dent Clin North Am. 2019;63(2):249–261. 10.1016/j.cden.2018.11.006 30825989

[3] Ender A , Zimmermann M , Mehl A . Accuracy of complete‐ and partial‐arch impressions of actual intraoral scanning systems in vitro. Int J Comput Dent. 2019;22(1):11–19. PMID: 30848250 30848250

[4] Paratelli A , Vania S , Gómez‐Polo C , Ortega R , Revilla‐León M , Gómez‐Polo M . Techniques to improve the accuracy of complete arch implant intraoral digital scans: a systematic review. J Prosthet Dent. 2023;129(6):844–854. 10.1016/j.prosdent.2021.08.018 34756427

[5] Papaspyridakos P , Bedrossian A , De Souza A , Bokhary A , Gonzaga L , Chochlidakis K . Digital workflow in implant treatment planning for terminal dentition patients. J Prosthodont. 2022;31(6):543–548. 10.1111/jopr.13510 35343618

[6] Ahmed WM , Alhazmi A , Alharbi MT , Azhari AA , Alqarni H , Shaheen HF , et al. Maxillary and mandibular complete‐arch implant rehabilitation using a complete digital workflow: a case report. J Prosthodont. 2023;32(8):662–668. 10.1111/jopr.13677 36905084

[7] Morton D , Gallucci G , Lin WS , Pjetursson B , Polido W , Roehling S , et al. Group 2 ITI Consensus Report: Prosthodontics and Implant Dentistry. Clin Oral Implants Res. 2018;29(16):215–223. 10.1111/clr.13298 30328196

[8] Papaspyridakos P , Bedrossian A , Kudara Y , Ntovas P , Bokhary A , Chochlidakis K . Reverse scan body: a complete digital workflow for prosthesis prototype fabrication. J Prosthodont. 2023;32(5):452–457. 10.1111/jopr.13664 36779654

[9] Khaohoen A , Powcharoen W , Sornsuwan T , Chaijareenont P , Rungsiyakull C , Rungsiyakull P . Accuracy of implant placement with computer‐aided static, dynamic, and robot‐assisted surgery: a systematic review and meta‐analysis of clinical trials. BMC Oral Health. 2024;24(1):359 10.1186/s12903-024-04033-y 38509530 PMC10956322

[10] Zhou W , Liu Z , Song L , Kuo CL , Shafer DM . Clinical factors affecting the accuracy of guided implant surgery‐a systematic review and meta‐analysis. J Evid Based Dent Pract. 2018;18(1):28–40. 10.1016/j.jebdp.2017.07.007 29478680

